# Comparative Metabolomic Study of *Drosophila* Species with Different Lifespans

**DOI:** 10.3390/ijms222312873

**Published:** 2021-11-28

**Authors:** Dmitry L. Maslov, Nadezhda V. Zemskaya, Oxana P. Trifonova, Steven Lichtenberg, Elena E. Balashova, Andrey V. Lisitsa, Alexey A. Moskalev, Petr G. Lokhov

**Affiliations:** 1 Analytical Branch, Institute of Biomedical Chemistry, 10 Building 8, Pogodinskaya Street, 119121 Moscow, Russia; oxana.trifonova@gmail.com (O.P.T.); sl@metabometrics.com (S.L.); balashlen@mail.ru (E.E.B.); lisitsa060@gmail.com (A.V.L.); lokhovpg@rambler.ru (P.G.L.); 2 Laboratory of Geroprotective and Radioprotective Technologies, Komi Science Center, Institute of Biology, Russian Academy of Sciences, 167982 Syktyvkar, Russia; kukushonok90@yandex.ru (N.V.Z.); amoskalev@list.ru (A.A.M.); 3 Metabometrics Inc., 651 N Broad Street, Suite 205 #1370, Middletown, DE 19709, USA

**Keywords:** metabolomics, metabolome profiling, direct mass spectrometry, longevity, aging, fruit fly, *Drosophila*

## Abstract

The increase in life expectancy, leading to a rise in the proportion of older people, is accompanied by a prevalence of age-related disorders among the world population, the fight against which today is one of the leading biomedical challenges. Exploring the biological insights concerning the lifespan is one of the ways to provide a background for designing an effective treatment for the increase in healthy years of life. Untargeted direct injection mass spectrometry-based metabolite profiling of 12 species of *Drosophila* with significant variations in natural lifespans was conducted in this research. A cross-comparison study of metabolomic profiles revealed lifespan signatures of flies. These signatures indicate that lifespan extension is associated with the upregulation of amino acids, phospholipids, and carbohydrate metabolism. Such information provides a metabolome-level view on longevity and may provide a molecular measure of organism age in age-related studies.

## 1. Introduction

The increase in the proportion of older people in the population is a demographic trend that is increasing over time [1]. The considerable growth in life expectancy is accompanied by the prevalence of age-associated chronic diseases, many of which are the top causes of death in developed countries (heart diseases, cancer, neurodegenerative diseases, etc.) [2]. The development of effective treatments focused on delaying the progression of age-related disorders has significant medical and economic benefits at both social (reducing the healthcare expenditures, etc.) and personal levels (improving quality of life and health, etc.) [3]. The detection and exploration of the biological pathways that are strongly linked with the regulation of lifespan are effective approaches for understanding the aging aspects [4]. The uncovering of their underlying molecular mechanisms can provide important insights for the design of a therapeutic strategy for slowing the aging process, preventing the development of age-related disorders, and lengthening the healthy years of life [4].

Life expectancy is determined by a complex interaction of multiple factors (from genetic to numerous environmental factors) [5]. The basic “omics” sciences (genomics, transcriptomics, proteomics, and metabolomics) can provide comprehensive information on changes in the organism at multiple levels (from genes to low-molecular-weight substances) [6]. However, among all the “omics,” metabolomics is perhaps the most relevant for describing the underlying mechanisms of biological processes. The metabolome is the final downstream product of cascades biological events resulting from a complex interplay of the genes, protein expressions, and the various environmental exposures (lifestyle, diet, gut microbial activity, etc.) [7]. Thus, metabolic alterations can serve as an unbiased source for generating hypotheses about changes in upstream control processes on multiple levels of systemic regulation (genomic, transcriptional, and proteomic levels), making metabolomics particularly relevant to the investigation of various biological processes [8,9,10]. Discovery and analysis of regulatory relationships between variations in metabolomic composition and diversity of lifespan can provide a comprehensive understanding of the physiological mechanisms of longevity [11,12]. Notably, in recent years, panoramic metabolite profiling has emerged as a powerful tool that has been widely and successfully applied in the investigation of biological processes, including the molecular patterns associated with life extension in non-mammalian and mammalian models [7,8,12].

The similarities between many metabolic pathways in flies and humans make *Drosophila* (different species of drosophilids) one of the attractive models for various biomedical studies (including longevity related) [13,14,15]. The strengths of *Drosophila* as a model are the relatively short lifespan, high fecundity, the simplicity of maintenance and cultivation, the fully annotated genome, the lack of regulatory ethical guidelines for experiments with invertebrate animals, etc. [14,16,17]. In addition, the availability of a fly species with significant differences in natural life expectancy is a notable advantage for longevity-related studies.

**Figure 1:**
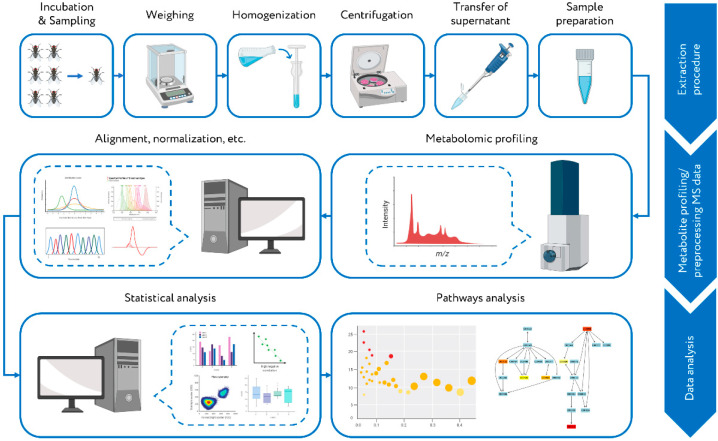
Workflow of the metabolomics study of fly species with different lifespans. Twelve different *Drosophila* species were cultivated in the fly incubator. Sampling was carried out every 2–6 days. The collected samples (whole adult fly samples) were divided into three biological replicates, weighed, and homogenized (methanol:water 4:1). After centrifugation, the supernatant was submitted to metabolite profiling by high-resolution direct mass spectrometry (MS). The data processing of obtained MS data (alignment, normalization, etc.) was performed. Further data analysis (univariate and multivariate statistical analysis) revealed the relationships between the metabolomic composition of various groups of samples and allowed to detect metabolites with statistically significant variations between compared groups. The retrieved results were used for the over-represented metabolite set analysis to interpret the data at the metabolic pathway level.

The untargeted direct-injection mass spectrometry (DIMS)-based metabolite profiling of 12 species of *Drosophila* with significant variations in natural lifespan was used in this study (Figure 1). A cross-comparison study of metabolomic profiles revealed the molecular signatures associated with the difference in life expectancy. At present, the number of panoramic omics-level studies providing comprehension of regulatory mechanisms of longevity is limited [18]. The conducted metabolomic analysis of flies can enrich this knowledge as some obtained results may be important for a more profound understanding of the regulatory mechanisms of lifespan at the molecular level.

## 2. Results

### 2.1. Drosophila Cohorts and Mass Spectrometry Analysis

The design of the comparative study was based on the difference in lifespans across the *Drosophila* species and, from the literature, the existence of age-associated metabolic alterations [10,19,20,21,22]. At first, the *Drosophila* species were grouped into three cohorts according to their life expectancy (long-lived, medium-lived, and short-lived species). Then, the DIMS was used to profile the metabolic composition of the flies. Over 12,000 mass spectrometry (MS) peaks with the mass-to-charge ratio (*m*/*z*) of up to 1000 Da were detected by an untargeted analytical approach across the studied samples. Preliminary processing of the metabolic dataset allowed the selection of MS peaks that were presented in at least 75% of the samples in each species cohort. Only these MS peaks were submitted to further analysis.

**Figure 2:**
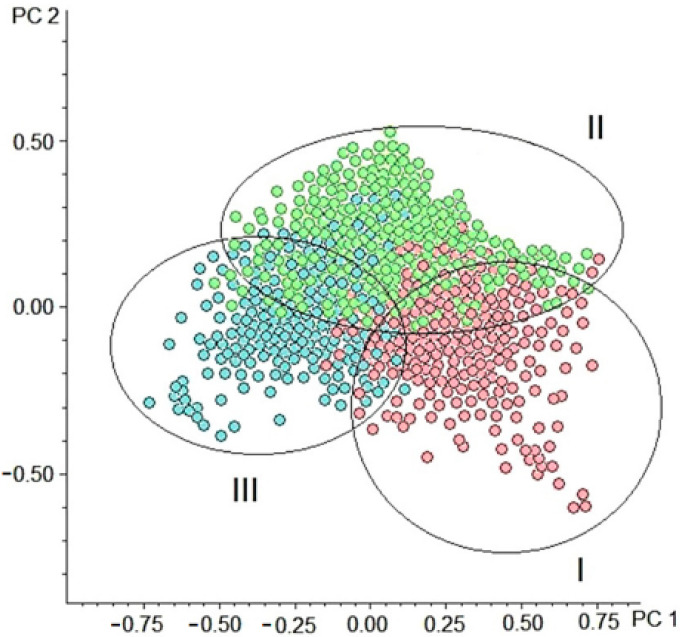
Principal component analysis (PCA) score plot (PC1 vs. PC2) of metabolomic profiling data. (●) Samples from the cohort of the long-lived species (group I), (●) samples from the cohort of the medium-lived species (group II), and (●) samples from the cohort of the short-lived species (group III). The first two principal components (PC1 and PC2) explain about 70% of the total variation in the spectra.

In the next stage of the data analysis, the overall difference in the metabolomic composition among the compiled cohorts was evaluated by the principal component analysis (PCA). The score plot displayed a grouping of fly samples according to the assigned cohort membership (Figure 2). This fact confirmed that the metabolome of *Drosophila* species is lifespan associated, which generally justifies the study's design based on lifespan-associated cohorts.

Then, the fly samples with equivalent age from each group were submitted to the cross-comparison study of metabolomic compositions. The fly sample selection provided a decrease in the influence of the age-associated alterations. The relative age was used as the criterion for the selection. The chronological ages (days) of all collected fly samples were transformed to relative values (Table S1).

### 2.2. Statistical Analysis and Metabolite Annotation

In the next stage of the study, a univariate analysis was performed. Non-parametric techniques were used due to violation of assumptions that should be met for the application of parametric tests (data not shown). The difference in metabolomic composition between cohorts was evaluated using a Kruskal–Wallis *H* test. The study revealed the complete list of *m*/*z* that are the source of heterogeneity between the cohorts of samples. A total of 318 MS peaks showed a significant change (*p* ≤ 0.05) between the cohorts. Among these MS peaks, 28 metabolites were putatively annotated. Annotation of 9 metabolites was confirmed by tandem mass spectrometry (MS/MS) (Table S2). Based on the results of annotation, the selected metabolites were grouped into several groups: carbohydrates, amino acids, carnitine, biogenic amine, and phospholipids. Subsequently, a pairwise comparison of the annotated metabolites between each cohort by Mann–Whitney *U* test was performed. Results of univariate analysis (Kruskal–Wallis *H* test and Mann–Whitney *U* test) are summarized in Table 1.

**Figure 3:**
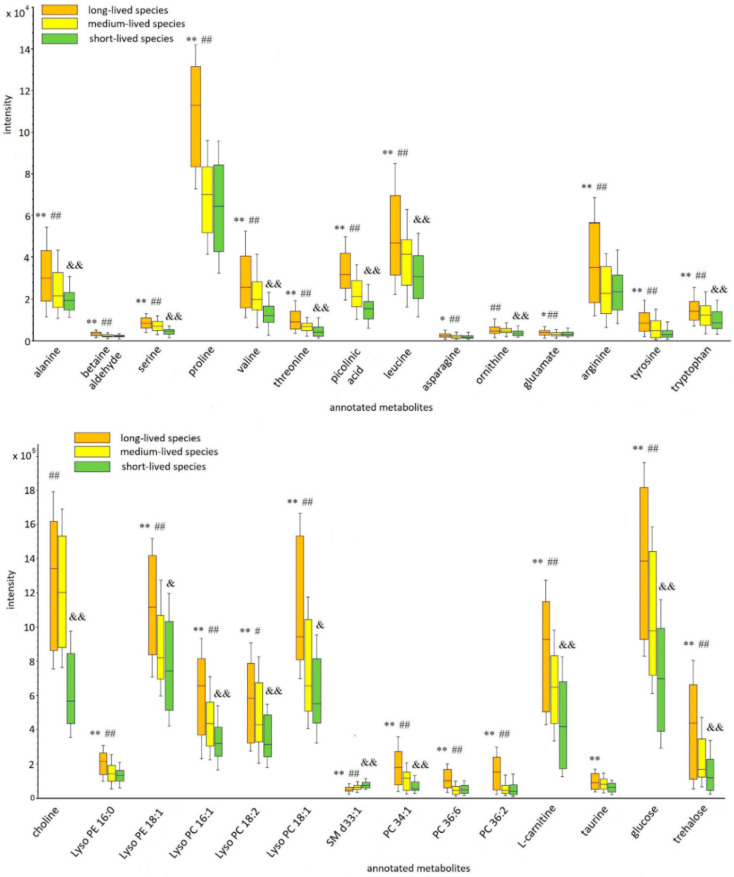
Box and whisker plots of the annotated metabolites between cohorts of *Drosophila* species. The box-whisker plot presents the distribution of normalized intensity values of the annotated metabolites. The top and bottom of the boxes represent the 25% and 75% percentiles; the 5% and 95% percentiles are indicated as error bars. The median value is indicated by horizontal lines within each box. The outliers were eliminated before the analysis. The results of the pairwise comparisons of the metabolite levels between cohorts are displayed (the changes were calculated using medians of individual metabolite levels across the lifespan). The pairwise differences were calculated by the Mann–Whitney *U* test (* *p* ≤ 0.05 (** *p* ≤ 0.01), significant change between long-lived and short-lived species; ^#^ *p* ≤ 0.05 (^##^ *p* ≤ 0.01), significant change between long-lived and medium species; ^&^ *p* ≤ 0.05 (^&&^ *p* ≤ 0.01), significant change between medium-lived and short-lived species).

The overview of the results of the cross-cohort analysis indicated that the highest level of the annotated metabolites was observed in the long-lived fly species and the lowest in the short-lived species. The exception was sphingomyelin (SM d33:1), the highest levels of which were in the samples of the short-lived species. A pairwise comparison of metabolite levels showed that all annotated metabolites differed significantly between the long- and short-lived cohorts. At the same time, there was no significant difference in the levels of some metabolites between long- and medium-lived or medium- and short-lived cohorts of species (Table 1). A possible relatively high similarity in the metabolomic composition of medium-lived species with species of both other compared cohorts is the reason for this. The variation in the levels of the annotated metabolites between the cohorts of species is illustrated in Figure 3.

### 2.3. Metabolic Pathway Enrichment Analysis

The metabolic pathway analysis (functional analysis module implemented in MetaboAnalyst v. 5.0, www.metaboanalyst.ca, accessed on 22 June 2021) was applied to detect the relevant metabolic pathways. The list of annotated metabolites was submitted into MetaboAnalyst to clarify the metabolic pathways associated with the differentially abundant metabolites. The analysis revealed seven enriched metabolic pathways. The metabolic pathway analysis plot is shown in Figure S1. The results of the analysis are summed in Table 2.

Thus, based on the result of the analysis, the following differentially abundant metabolic pathways were chosen as the significantly relevant pathways in terms of *p*-value and impact value: aminoacyl-tRNA biosynthesis; valine, leucine, and isoleucine biosynthesis; arginine biosynthesis (Figure S2); arginine and proline metabolism; alanine, aspartate, and glutamate metabolism; glycine, serine, and threonine metabolism; and starch and sucrose metabolism. The complete list of the enriched pathways and corresponding metabolites identified within the pathways is shown in Table S3.

### 2.4. Ontogenetic (Intra-Group) Analysis

**Figure 4:**
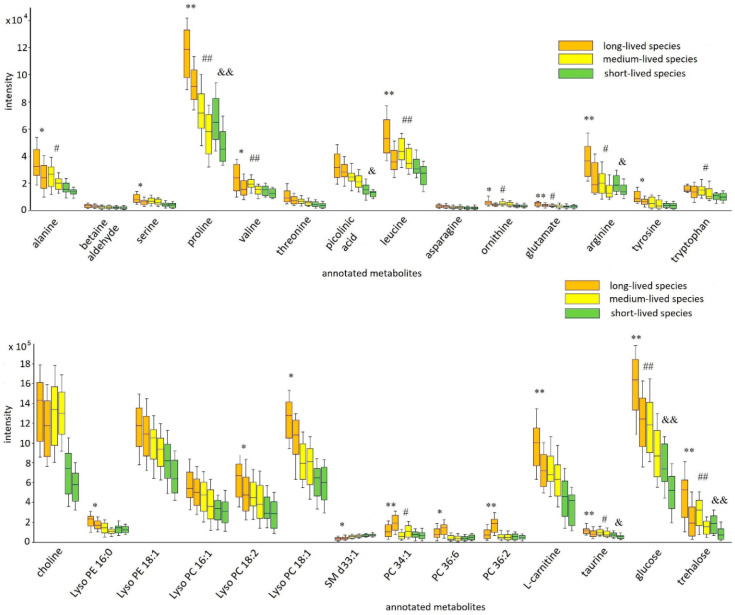
Box plot graphs of the age-related drift of selected metabolites within every fly cohort. The box-whisker plot presents the distribution of normalized intensity values of the annotated metabolites. Each cohort is represented by two boxes of the same background color. The first box (left box) is the young subgroup, and the second box (right box) is the mature subgroup. The top and bottom of the boxes represent the 25% and 75% percentiles; the 5% and 95% percentiles are indicated as error bars. The median value is indicated by horizontal lines within each box. In addition, the results of the pairwise comparisons of the metabolite levels between the two subgroups in each cohort are displayed (Table S2). The pairwise differences were calculated by the Mann–Whitney *U* test (* *p* ≤ 0.05 (** *p* ≤ 0.01), significant change between subgroups (young vs. mature) of long-lived species; ^#^ *p* ≤ 0.05 (^##^ *p* ≤ 0.01), significant change between subgroups (young vs. mature) of medium species; ^&^ *p* ≤ 0.05 (^&&^ *p* ≤ 0.01), significant change between subgroups (young vs. mature) of short-lived species).

In the next stage of the experiment, ontogenetic changes of the identified metabolites were discovered. Unfortunately, the absence of visible age-related phenotypic signs and objective methods for determining the age of adult fruit flies do not allow to clearly define the boundary of different age periods (young, adult, mature, elderly). The relative biological age was used as a criterion for the formation of the different age subgroups in each cohort (long-, medium-, and short-lived fly species). The first subgroup included the samples in which the relative biological age varied from 10 to 30% of the maximum lifespan of the species (the subgroup was named “young”). The second subgroup consisted of the samples in which the relative biological age was from 60 to 70% of the maximum lifespan of the species (the subgroup was named “mature”). The detailed information about the formed subgroups (the maximum lifespan of each species, the relative and chronological age of the samples included in the subgroups, etc.) is summarized in Supplementary Table S1. The ontogenetic alterations (young vs. mature) in each cohort were evaluated by the Mann–Whitney *U* test (results are summarized in Table S4). The variation in the levels of the annotated metabolites is illustrated in (Figure 4).

Results of the intra-cohort comparison (comparative analysis of the formed subgroups in each cohort) demonstrated a decrease in the levels of most annotated metabolites in all cohorts as animals mature. It should be noted that not all of the ontogenetic alterations were statistically significant, but a decline in the level of the annotated metabolites with age was observed in all cohorts. A small fraction of metabolites (that were putatively annotated as phospholipids: PC 34:1, PC 36:6, and PC 36:2), opposite, demonstrated the elevation of level with age in all formed cohorts (Figure 4). The pronounced changes in metabolite levels were detected among the young and mature subgroups of long-lived species. At the same time, in most cases, the subgroups of cohorts of short- and medium-lived species possessed no statistically significant age-related differences.

In the last stage of the experiment, cross-cohort comparative studies of the formed subgroups were performed (young vs. young; mature vs. mature). The analysis enabled the evaluation of variations in the levels of the annotated metabolites between cohorts at the different periods of the adult phase of the fly life cycle. The difference was evaluated by the Kruskal–Wallis *H* test. Subsequently, a test of pairwise differences of the selected metabolites between each cohort by Mann–Whitney *U* test was performed. The results of univariate analysis (the Kruskal–Wallis *H* test and the Mann–Whitney *U* test) are summarized in Supplemental Table (Table S5). Visualization of the results of the comparative analysis is presented in Figure S3.

Results of the cross-cohort comparative study displayed the availability of variations in abundance levels of the annotated metabolites both between the younger age subgroups and between the oldest age subgroups. The presence of the differences in the metabolite levels at the various stages of the adult phase is a confirmation of the correct choice of metabolites. Nevertheless, it should be noted that the most significant alterations in the metabolite abundance levels were found between the young subgroups of formed cohorts (the significant difference *p* ≤ 0.05). While, in many cases, there were no significant differences between the mature subgroups (*p* > 0.05) (Table S5).

## 3. Discussion

The design of the experiment to reveal the longevity signatures was based on the cross-comparison study of metabolomic compositions of species with different lifespans. Initially, it was assumed that the comparative analysis of the metabolomic composition of each fly species with every other fly species is hardly effective. Very likely, the source of most of the metabolomic variations between species is the result of phenotypic plasticity that is a consequence of evolutionary adaptation to prevailing environmental factors and not related to the longevity mechanism. It is complicated to isolate and identify from this metabolite set the particular metabolites involved in the lineage-specific lifespan extension mechanisms. It was decided to focus on studying common longevity-related mechanisms that apply across closely related species with a similar lifespan [18]. For this purpose, species with a similar lifespan were grouped into separate cohorts. This allowed eliminating the species-specific perturbations (only cohort-specific perturbations that are typically for all fly species of this cohort were enabled for analysis). The approach based on comparing the cohort-specific metabolic compositions minimizes the number of significantly different metabolites, which potentially correlates with the difference in longevity [8,23,24].

Due to the dramatic lifespan fluctuation across the species, individuals of different species with similar chronological ages are at various stages of life. In addition, the metabolomic composition of model organisms changes significantly throughout life [19,20,21,22]. In this case, the hallmarks of aging may be expected to have detectable effects on the metabolome that can prevent a correct selection of relevant metabolites. In turn, it can affect the interpretation of the outcome of the comparative study, leading to a reduction in the research effectiveness [25]. Considering the lack of clear parameters for the evaluation of adult fly aging, the relative age was proposed as the criterion. The relative age in every sampling point was expressed as a percentage of the maximum lifespan of the species (the maximum lifespan of the species was taken as 100%). Thus, the design of the study was based on the selection of samples of equivalent relative age from each species. Then, the selected samples were combined in the cohorts for the comparative analysis.

Results of the comparative analysis showed that significantly different metabolites belong to various chemical classes: carbohydrates, amino acids, carnitines, biogenic amines, and lipids. The highest level of most annotated metabolites was observed in long-lived species. Based on the list of annotated metabolites, the relevant enriched pathways were detected. Most of these pathways were associated with amino acid biosynthesis and metabolism. Despite numerous studies demonstrating the link of the amino acid level and life expectancy [26,27,28,29,30,31,32], the specific mechanisms of the amino acid-mediated modulation of the lifespan are little known [33]. Alterations in the amino acid level can lead to changes in the activity of the amino acid metabolism-related processes (intensification of protein synthesis and synthesis of biologically active compounds, amplification of cellular differentiation and growth, up-regulation of antioxidant defense and immune response systems, etc.), which may be one of the causes of the observed phenomenon [28,34,35,36,37]. The effect of amino acids on energy metabolism should be noted especially. The positive correlation between the enhancement of energy metabolism and lifespan extension was demonstrated in many species [38,39]. Earlier, it was shown that amino acids can modulate energy metabolism through enhancement of the mitochondrial biogenesis [36,40,41], via activation of the synthesis of various mitochondrial components (mitochondrial protein synthesis, etc.) [42], and by elevation of adenosine triphosphate (ATP) production (products of amino acid catabolism can enter into the TCA cycle as intermediates) [28].

Previously published studies have shown that the level of sugars (glucose and trehalose) has an important role in the regulation of the lifespan in such model organisms as worms and fruit flies (modest elevation in their levels promoted an extending of life) [43,44,45]. These sugars have a wide range of biological effects. On one hand, the sugars are the main circulating fuel sources and precursors of various building blocks for cellular biosynthesis [46,47,48]. On the other hand, they are involved in the regulation of various processes (autophagy, maintenance of water homeostasis, immunity, etc.) [45,49,50,51]. Probably, a combination of the beneficial effects of the sugars is the reason for the positive effect on longevity processes. The relevance of sugars to longevity can also be explained by the ratio of proteins to carbohydrates (P:C), which affects the lifespan of flies [52]. The lowering of this ratio leads to a lifespan extension [53,54]. Based on this phenomenon, it has been hypothesized that high sugar levels in long-lived species lead to a drop in the (P:C) ratio and thus increase longevity [45]. However, despite numerous studies on the (P:C) ratio in prolonging lifespan, the mechanism of this phenomenon is still being debated [55]. One of the possible mechanisms is upregulation in the expression of antimicrobial peptides, leading to improved immunity and resistance to infection [56].

The last group of metabolites that are the source of heterogeneity among the comparative species is lipids (Table 1). There were no enriched lipid-associated pathways in the study. Probably, lipidomics-based approaches should be used for the identification of the specific lipid-related pathways. The potential mechanisms of the influence of lipids on life expectancy and the role of particular lipids in the process are still poorly understood [57,58,59]. Nevertheless, a close link between alterations in cellular lipid composition and lifespan has been demonstrated in numerous studies [57]. Apparently, the impact of lipid composition on life expectancy is associated with regulation of the effectiveness of some cellular processes (autophagic activity, etc.) and physical properties of the membrane of cellular organelles (the viscosity of mitochondrial membrane, etc.) [57]. The observed versatile change in lipid levels with age may reflect age-related changes in the lipid ratios of cell membranes.

Thus, based on the obtained results, it was hypothesized that the longevity in fly species was associated with shifts in amino acid, carbohydrate, and lipid metabolism. A review of the biological effects of annotated metabolites indicated that a common attribute in most is the ability to integrate into the regulation of cellular energy production (as fuel sources, modulating factors, etc.). Energy metabolism plays a crucial role in a plethora of vital cellular processes [60]. A decrease in the energy supply or any failures in the cellular energy status leads to a significant reduction in lifespan [61]. Probably, a shift in energy production is one of the specific patterns of long-lived species. Thus, the enhancement of cell energy metabolism in combination with beneficial effects associated with the annotated metabolites and metabolite-related pathways is one of the potential longevity-related mechanisms.

The found alterations in metabolite levels as animals mature can indicate their possible link with the aging process. A review of the trajectories of the annotated metabolite levels changes showed that they resemble the age-related alterations in metabolites identified in the previously published studies [62,63]. Remarkably, at least some of the annotated metabolites (trehalose, BCAA) are involved in the earlier discovered metabolic signatures related to aging [62]. It was hypothesized that longevity and aging processes are similar and can be regulated by the same metabolic pathways. Perhaps, the lifespan extension of long-lived species is determined by genetics-based up-regulation of the annotated metabolite that leads to enhancement of beneficial effects of detected metabolite-associated pathways.

The application of the omics-based approaches revealed the relevant molecular pathways associated with metabolites of interest. It was hypothesized that these pathways are potentially associated with longevity and aging processes and the variations in their biological activities contribute to the interspecific difference in life expectancy. The background can provide a design of molecular signatures of biological age. The metabolomics-based signatures can be used as a tool for developing a clock of age. Previous studies have shown a strong correlation of metabolic profiles with age [64,65]. Thus, the model for assessing the health and functional capacity of an organism based on metabolomic signatures has a great future. The enriched pathways should also be studied in other species, including mammals. We can hope that due to the similar basic biological processes of flies and mammals, at least some of the detected pathways associated with the regulation of life expectancy in flies can be revealed in mammals. The knowledge can contribute to the understanding of the general principles of lifespan control on the molecular level and can help pave the way for the design of new therapeutic strategies that may promote the modest extension of the healthy years of life.

## 4. Materials and Methods

### 4.1. *Drosophila* Maintenance

The living cultures of 12 different species of Drosophilid, *D. virilis*, *D. ananassae*, *D. saltans*, *D. simulans*, *D. austrosaltans*, *D. bipectinata*, *D. yakuba*, *D. melanogaster*, *D. willistoni*, *D. erecta*, *D. kikkawai*, and *D. biarmipes*, were purchased from UC San Diego Stock Center (La Jolla, CA, USA). The species were grouped into the 3 groups according to their lifespan (long-lived, medium-lived, and short-lived species). General information about the species of flies is presented in Table 3.

The flies were maintained in the fly incubator at 25 °C and 60% humidity in a 12 h/12 h light/dark cycle on a standard sugar-yeast-based diet (7 g of agar, 30 g of sugar, 30 g of semolina, 8 g of yeast, and 10 mL/L propionic acid) [84]. The day of emerging from the pupae was defined as day 1 for each species. The samples for analysis were obtained every 2–6 days. At each time point, flies from each of the 12 species were gently collected (about 30 male adult flies from each of the species) and segregated into 3 equal fractions (each about 10 flies). Every fraction of flies was transferred to individual Eppendorf, frozen, and stored at −80 °C. The sampling was stopped when 80 percent of the flies of a particular species died (the surviving animals were not representative of the species population).

### 4.2. Sample Preparation

Every collected fraction (about 10 whole adult flies) was segregated into two or three equal sub-fractions (biological replicates) and weighed. Next, the flies of each sub-fractions were defrosted on ice and accurately washed to remove food, debris, and other types of soil from the body surface. For this purpose, the flies were placed into centrifuge tube filters (Corning Costar Spin-X, Corning Life Sciences, NY, USA). Then 1 mL of ice water (Sigma-Aldrich, St. Louis, MO, USA) was added to the samples and gently vortexed for 1 min, followed by centrifugation for 15 min at 800× *g*, 4 °C (Centrifuge 5804R; Eppendorf AG, Hamburg, Germany). The washing procedure was repeated twice. The extraction was performed according to [85,86,87] with slight modifications. The pre-weighed sub-fractions were transferred to a pre-cooled 1 mL glass hand-held homogenizer (Kimble-Chase, Rockwood, TN, USA) containing a mixture of methanol:water (4:1, *v*/*v*; 28.5 μL/mg (dry mass)). The samples (whole flies) were homogenized on ice three times for 2–3 min at 3- to 4-min intervals (homogenizer was maintained on ice and periodically vortexed). The total time of extraction was 15 min. The obtained homogenate was transferred into the pre-cooled glass centrifuge tube. Then, an equal volume of the same mixture was added to the homogenizer, vortexed for 1 min, and transferred into a centrifuge tube with homogenate. This step enabled collecting the remaining homogenate particles from the homogenizer and glass pestle. Thus, the final volume of the mixture methanol:water (4:1, *v*/*v*) was 57 μL/mg (dry mass). After centrifugation (15,000× *g*, 4 °C, 15 min), the supernatant was transferred to a clean 200 µL glass vial (Waters, Milford, MA, USA), which was placed into a glass autosampler vial (1.5 mL) with a screw cap (Waters, Milford, MA, USA). A blank sample containing only the mixture of methanol:water (4:1, *v*/*v*) was prepared in parallel.

Before the mass spectrometry analysis, an aliquot (10 µL) of the supernatant was diluted 50-fold by 90% methanol (J.T. Baker, Gliwice, Poland) with 0.1% formic acid (Fluka, Munich, Germany) [88,89,90]. As an internal standard, 0.4 µL (5 mg/L) of losartan solution was added.

All solvents were of HPLC and UHPLC grade: water (Sigma-Aldrich, St. Louis, MO, USA), methanol (J.T. Baker, Gliwice, Poland), and formic acid (Fluka, Munich, Germany).

### 4.3. Metabolite Profiling

Mass spectrometry analysis of the metabolite composition was performed using a hybrid quadrupole time-of-flight mass spectrometer (maXis Impact, Bruker Daltonics, Billerica, MA, USA) equipped with electrospray ionization (ESI). The targeted scan range of *m*/*z* 50–1000 was applied, with mass accuracy up to 3 parts per million (ppm). Mass calibration was performed daily before beginning the analysis, set by use of external calibration standard ES Tuning Mix (Agilent Technologies, Santa Clara, CA, USA). A glass syringe (Hamilton Bonaduz AG, Bonaduz, Switzerland) and a syringe injection pump (KD Scientific, Holliston, MA, USA) with a flow rate of 180 µL/h were used for direct injection of analyte into the ESI source [88,89]. Mass spectra were recorded by DataAnalysis software (version 3.4, Bruker Daltonics, Bremen, Germany) to summarize signals for 1 min. Three technical replicates per sample were performed.

### 4.4. Preprocessing MS Data

The MS raw data generated by the experiments were processed using DataAnalysis software and converted into a peak list. The peak lists were generated with the following parameters: peak width, 3; signal-to-noise ratio, 2; and relative and absolute threshold intensity, 0.05% and 100. A recalibration procedure carried out using reference masses proved the achievement of high mass measurement accuracy. Normalization of mass peak intensities and data filtration to eliminate low-informative peaks were carried out using the self-made algorithm [91]. Peaks, detected in various samples, were related to the same feature ion if the mass difference did not exceed ±0.01 Da.

### 4.5. Metabolite Annotation

Annotation of the mass spectrometry peaks with a clear isotope pattern was carried out manually by comparing the *m*/*z* value of the feature and their isotopic distribution with annotated metabolites in the public metabolite databases: Human Metabolome Database (HMDB) (www.hmbd.ca, accessed on 25 May 2021), METLIN (http://metlin.scripps.edu, accessed on 25 May 2021), and LIPID MAPS (http://lipidmaps.org, accessed on 12 May 2021). Mass tolerance was 0.01 Da. A theoretical isotope pattern was produced using Isotope Pattern Calculator (Bruker Daltonics, Bremen, Germany). The algorithm of metabolite annotation based on two orthogonal characteristics (accurate mass and isotopic distribution) was satisfied to level 2 identification (putatively annotated compounds), according to the Metabolomics Standards Initiative (MSI) guidelines [92].

Lipid annotation variants were suggested according to accurate mass measurements only. The structure of acyl chains was not elucidated. The identification met level 3 (putatively annotated compound classes) according to MSI guidelines. The tandem mass spectrometry (MS/MS) approach was applied for more robust identification of selected metabolites. In this case, identification was achieved by comparing experimental MS/MS spectra obtained at different collision energies in positive ionization mode with the MS/MS fragmentation patterns from the public metabolite databases (HMDB, METLIN).

### 4.6. Data Analysis

Multivariate statistical analysis of the metabolite profiling data (by principal component analysis (PCA)) were carried out in ProfileAnalysis (Bruker Daltonics, Billerica, MA, USA). Univariate statistical analysis (non-parametric Kruskal–Wallis *H* test, pairwise Mann–Whitney *U* test) was performed using Statistica software 10.0 (StatSoft Inc., Tulsa, OK, USA), with *p* ≤ 0.05 set as the level of statistical significance. The MetPA (metabolic pathway analysis) web-based tool implemented in MetaboAnalyst 5.0 software was used to determine the relevant metabolic pathways that are most different between the species groups. The fruit fly (*Drosophila melanogaster*) library from the KEGG database and hypergeometric test were selected as options for the analysis algorithm to identify the metabolic pathways. The impact values over 0.1 and the *p*-values lower than 0.05 were taken as the thresholds [93].

## Figures and Tables

**Table 1:** List of the significantly different (*p* ≤ 0.01) metabolites between the cohorts of fly species.

No.	Metabolite	Measured *m*/*z*	Calculated *m*/*z*	Ion Form	Elemental Composition	*p*-Value			
						*H* Test	*U* Test Long-Lived vs. Short-Lived	*U* Test Long-Lived vs. Medium-Lived	*U* Test Medium-Lived vs. Short-Lived
1	Alanine	90.0545	90.0550	[M + H]^+^	C_3_H_7_NO_2_	5 × 10^−5^	1 × 10^−6^	9 × 10^−4^	6 × 10^−3^
2	Betaine aldehyde	102.0911	102.0913	[M]^+^	C_5_H_12_NO	2 × 10^−5^	5 × 10^−7^	7 × 10^−5^	2 × 10^−1^
3	Serine	106.0501	106.0498	[M + H]+	C_3_H_7_NO_3_	1 × 10^−5^	1 × 10^−7^	2 × 10^−4^	3 × 10^−6^
4	Proline	116.0709	116.0705	[M + H]^+^	C_5_H_9_NO_2_	3 × 10^−4^	9 × 10^−7^	2 × 10^−6^	3 × 10^−1^
5	Valine *	118.0867	118.0862	[M + H]^+^	C_5_H_11_NO_2_	9 × 10^−4^	4 × 10^−6^	6 × 10^−3^	2 × 10^−5^
6	Threonine	120.0658	120.0655	[M + H]^+^	C_4_H_9_NO_3_	5 × 10^−4^	2 × 10^−7^	7 × 10^−5^	3 × 10^−6^
7	Picolinic acid *	124.0392	124.0392	[M + H]^+^	C_6_H_5_NO_2_	8 × 10^−5^	2 × 10^−6^	8 × 10^−5^	1 × 10^−6^
8	Leucine *	132.1024	132.1018	[M + H]^+^	C_6_H_13_NO_2_	5 × 10^−5^	4 × 10^−6^	5 × 10^−4^	7 × 10^−4^
9	Asparagine	155.0450	155.0430	[M + Na]^+^	C_4_H_8_N_2_O_3_	5 × 10^−3^	4 × 10^−3^	9 × 10^−3^	4 × 10^−1^
10	Ornithine	155.0811	155.0791	[M + Na]^+^	C_5_H_12_N_2_O_2_	2 × 10^−4^	2 × 10^−4^	6 × 10^−1^	1 × 10^−4^
11	Glutamate *	170.0420	170.039	[M + Na]^+^	C_5_H_9_NO_4_	2 × 10^−3^	2 × 10^−2^	8 × 10^−4^	9 × 10^−1^
12	Arginine *	175.1201	175.1191	[M + H]^+^	C_6_H_14_N_4_O_2_	1 × 10^−4^	1 × 10^−4^	4 × 10^−6^	8 × 10^−1^
13	Tyrosine	182.0812	182.0812	[M + H]^+^	C_9_H_11_NO_3_	9 × 10^−4^	3 × 10^−6^	2 × 10^−6^	1 × 10^−1^
14	Tryptophan *	205.0961	205.0971	[M + H]^+^	C_11_H_12_N_2_O_2_	3 × 10^−5^	2 × 10^−6^	8 × 10^−3^	5 × 10^−3^
15	Choline	104.1068	104.1069	[M]^+^	C_5_H_14_NO	7 × 10^−5^	1 × 10^−6^	5 × 10^−1^	4 × 10^−6^
16	Lyso PE 16:0 **	454.2926	454.2928	[M + H]^+^	C_21_H_44_NO_7_P	4 × 10^−4^	2 × 10^−6^	6 × 10^−6^	3 × 10^−1^
17	Lyso PE 18:1 **	480.3078	480.3085	[M + H]^+^	C_23_H_46_NO_7_P	9 × 10^−5^	8 × 10^−6^	2 × 10^−5^	2 × 10^−2^
18	Lyso PC 16:1 **	494.3237	494.3241	[M + H]^+^	C_24_H_48_NO_7_P	5 × 10^−5^	8 × 10^−7^	1 × 10^−5^	7 × 10^−5^
19	Lyso PC 18:2 **	520.3388	520.3398	[M + H]^+^	C_26_H_50_NO_7_P	4 × 10^−4^	2 × 10^−5^	3 × 10^−2^	5 × 10^−5^
20	Lyso PC 18:1 **	522.3550	522.3554	[M + H]^+^	C_26_H_52_NO_7_P	1 × 10^−5^	3 × 10^−8^	2 × 10^−6^	3 × 10^−3^
21	SM d33:1 **	689.5586	689.5592	[M + H]^+^	C_38_H_77_N_2_O_6_P	3 × 10^−5^	4 × 10^−7^	3 × 10^−6^	2 × 10^−4^
22	PC 34:1 **	760.5839	760.5843	[M + H]^+^	C_42_H_82_NO_8_P	1 × 10^−6^	2 × 10^−7^	1 × 10^−6^	8 × 10^−4^
23	PC 36:6 **	778.5368	778.5381	[M + H]^+^	C_44_H_76_NO_8_P	7 × 10^−4^	1 × 10^−5^	3 × 10^−5^	1 × 10^−1^
24	PC 36:2 **	786.5998	786.6007	[M + H]^+^	C_44_H_84_NO_8_P	8 × 10^−5^	2 × 10^−6^	6 × 10^−6^	4 × 10^−1^
25	Carnitine *	162.1126	162.1124	[M + H]^+^	C_7_H_15_NO_3_	5 × 10^−6^	1 × 10^−7^	7 × 10^−6^	1 × 10^−5^
26	Taurine	148.0044	148.0039	[M + Na]^+^	C_2_H_7_NO_3_S	6 × 10^−4^	1 × 10^−5^	5 × 10^−2^	5 × 10^−2^
27	Glucose *	203.0545	203.0526	[M + Na]^+^	C_6_H_12_O_6_	1 × 10^−6^	7 × 10^−7^	1 × 10^−6^	1 × 10^−6^
28	Trehalose *	365.1085	365.1054	[M + Na]^+^	C_12_H_22_O_11_	4 × 10^−4^	2 × 10^−6^	6 × 10^−4^	8 × 10^−5^

*H* test, Kruskal–Wallis *H* test; *U* test, Mann–Whitney *U* test. * Identification of these metabolites was confirmed by the tandem mass spectrometry (MS/MS) approach. ** The exact structure of the acyl chains was not established for phospholipids and was suggested from database search results.

**Table 2:** Over-representation analysis (ORA) results for altered metabolites revealed in the comparative analysis of *Drosophila* species with different lifespans.

No.	Pathway Name ^1^	Total	Hits	Raw *p* ^2^	−log(*p*)	Impact
1	Aminoacyl-tRNA biosynthesis	48	12	**1.64 × 10^−8^**	11.8	0.17
2	Valine, leucine, and isoleucine biosynthesis	8	3	**0.0003**	3.5	0.13
3	Arginine biosynthesis	12	3	**0.0011**	2.9	0.63
4	Arginine and proline metabolism	31	4	**0.0020**	2.7	0.42
5	Alanine, aspartate, and glutamate metabolism	23	3	**0.0079**	2.1	0.28
6	Glycine, serine, and threonine metabolism	30	3	**0.0170**	1.8	0.32
7	Starch and sucrose metabolism	14	2	**0.0270**	1.6	0.17
8	Glyoxylate and dicarboxylate metabolism	24	2	0.0720	1.2	0.07

^1^ Pathways were sorted according to the probability (*p*-value) of detection of a particular number of significantly altered metabolites in the compound list of a certain pathway. ^2^ *p*-Values lower than 0.05 are marked by bold.

**Table 3:** Biological characteristics of the 12 fruit fly species.

Scientific Name	Genus/ Subgenus/Species Group/Species	Maximum Lifespan ^1^ (Days)	Native Habitancy ^2^	Development Time ^3^ (Days)	Body Length ^4^ (mm)	Natural Nutrition	Reference Genome/Stock Number
**Cohort** #**1** Long-lived species (aver. lifespan of animals (days) ± s.d. = 82.0 ± 25.4)							
*Drosophila* *ananassae*	*Drosophila/Sophophora/melanogaster/ananassae*	71	South, Southeast Asia, Polynesia, regions of Australia and Americas [66]	13	2.4 ± 0.03	Decaying fruits [67]	Ensembl dana_caf1.21/ 14024-0371.13
*Drosophila* *saltans*	*Drosophila/Sophophora/saltans/saltans*	70	Tropical North America [68]	17	2.6 ± 0.02	Rotting tropical fruits [69]	Unpublished/ 14045-0911.00
*Drosophila* *willistoni*	*Drosophila/Sophophora/willistoni/willistoni*	67	Central and South America, the Caribbean [70]	15.5	2.3 ± 0.02	Rotting fruits [70]	Ensembl dwil_caf1.21/ 14030-0811.24
*Drosophila* *virilis*	*Drosophila/Drosophila/virilis/virilis*	120	Deciduous forests of China, arid regions of Iran and Afghanistan [71]	20	3.6 ± 0.03	Fluxes of willows and other decaying parts of trees [72]	Ensembl dvir_caf1.21/ 15010-1051.87
**Cohort** #**2** Medium-lived species (aver. lifespan of animals (days) ± s.d. = 43.4 ± 5.9)							
*Drosophila* *austrosaltans*	*Drosophila*/ *Sophophora*/ *saltans/austrosaltans*	40	Central and South America [73]	16.5	2.7 ± 0.03	Rotting tropical fruits [69]	Unpublished/ 14045-0881.00
*Drosophila* *bipectinata*	*Drosophila*/*Sophophora*/ *melanogaster*/*bipectinata*	42	Southeast Asia, islands of Pacific Ocean [74]	14.5	1.6 ± 0.02	Fruits [75]	NCBI AFFE00000000.2/ 14024-0381.19
*Drosophila* *melanogaster*	*Drosophila*/*Sophophora*/ *melanogaster*/*melanogaster*	50	West Africa [76]	13	2.4 ± 0.04	Rotten, fermenting fruits; marula fruit [76]	Ensembl BDGP5.75/ 14021-0231.36
*Drosophila* *simulans*	*Drosophila*/*Sophophora*/ *melanogaster/simulans*	36	Sub-Saharan Africa, Madagascar [77]	12.5	2.8 ± 0.03	Rotting fruits [78]	Ensembl WUGSC1.21/ 14021-0251.194
*Drosophila* *yakuba*	*Drosophila*/*Sophophora*/ *melanogaster/yakuba*	49	Tropical Africa, Madagascar [76]	12.5	2.2 ± 0.02	Generalist fruit breeder [70]	Ensembl dyak_r1.3_FB2008_07.21/14021-0261.01
**Cohort** #**3** Short-lived species (aver. lifespan of animals (days) ± s.d. = 28.0 ± 2.6)							
*Drosophila* *erecta*	*Drosophila*/*Sophophora*/ *melanogaster/erecta*	25	Equatorial West Africa [79]	14.5	2.2 ± 0.02	Pandanus fruits [78]	Ensembl dere_caf1.21/ 14021-0224.01
*Drosophila* *biarmipes*	*Drosophila*/*Sophophora*/ *melanogaster/biarmipes*	30	Hindustan [80]	17.5	2.4 ± 0.03	Rotting fruit [81]	NCBI AFFD00000000.2/ 14023-0361.09
*Drosophila* *kikkawai*	*Drosophila*/*Sophophora*/ *melanogaster/kikkawai*	29	Hindustan, Brazil [82]	15.5	2.4 ± 0.02	Exotic fruits [83]	NCBI AFFH00000000.2/ 14028-0561.14

^1^ A vivarium was used for growth. ^2^ The original habitat area is specified. Currently, some species have a worldwide distribution, probably due to human movements and activities. ^3^ Development time (from egg to adult; 18 °C, days). Information was received from [73]. ^4^ Body length is for adult male fly (mean ± s.d.).

## Data Availability

The data presented in this study are available on request from the corresponding author.

## Supplementary Materials

The following are available online at https://www.mdpi.com/article/10.3390/ijms222312873/s1.
