# Supplementary material for: Comparative Metabolomic Study of Drosophila Species with Different Lifespans
Source: Int J Mol Sci. 2021 Nov 28;22(23):12873. doi: 10.3390/ijms222312873 (PMC8657752; doi:10.3390/ijms222312873)
Supplement: Supplementary file 1 [file ijms-22-12873-s001.zip › ijms-1452981-supplementary.pdf]

Supplementary Materials

Table S1. Distribution of the fly species between cohorts according to their lifespan.

	**Long-lived species**								**Medium-lived species**										**Short-lived species**					
	D.ananassae		*D.saltans*		*D.willistoni*		*D. virilis*		*D.austrosaltans*		*D.bipectinata*		*D.melanogaster*		*D.simulans*		*D.yakuba*		*D.biarmipes*		*D.erecta*		*D.kikkawai*	
	chronological age (days)	relative age (%)	chronological age (days)	relative age (%)	chronological age (days)	relative age (%)	chronological age (days)	relative age (%)	chronological age (days)	relative age (%)	chronological age (days)	relative age (%)	chronological age (days)	relative age (%)	chronological age (days)	relative age (%)	chronological age (days)	relative age (%)	chronological age (days)	relative age (%)	chronological age (days)	relative age (%)	chronological age (days)	relative age (%)
	1	1%	1	1%	1	1%	1	1%	1	3%	1	2%	1	2%	1	3%	1	2%	1	3%	1	4%	1	3%
	8	11%	8	11%	8	12%	11	9%	4	10%	4	10%	4	8%	4	11%	4	8%	4	13%	4	16%	4	14%
	17	24%	17	24%	17	25%	30	25%	11	28%	11	26%	14	28%	8	22%	14	29%	8	27%	8	32%	8	28%
	26	37%	26	37%	24	36%	44	37%	14	35%	14	33%	17	34%	14	39%	17	35%	11	37%	8	32%	11	38%
	30	42%	30	43%	28	42%	50	42%	17	43%	17	40%	22	44%	14	39%	20	41%	11	37%	11	44%	11	38%
	36	51%	36	51%	34	51%	60	50%	20	50%	22	52%	26	52%	17	47%	24	49%	14	47%	14	56%	14	48%
	44	62%	44	63%	40	60%	75	63%	24	60%	26	62%	30	60%	22	61%	30	61%	17	57%	14	56%	17	59%
	48	68%	48	69%	44	68%	80	67%	26	65%	28	67%	34	68%	24	67%	32	65%	20	67%	17	68%	20	69%
	50	70%	50	71%	48	72%	85	71%	28	70%	30	71%	36	72%	26	72%	34	69%	20	67%	17	68%	20	69%
maximal lifespan	71	100%	70	100%	67	100%	120	100%	40	100%	42	100%	50	100%	36	100%	49	100%	30	100%	25	100%	29	100%

The distribution of the fly species between three cohorts according to maximum lifespan is displayed. The maximum lifespan of each species is indicated at the end of the column. The age of each selected sample is presented both as chronological age (expressed in days) and relative age (expressed as a percentage to the maximum lifespan of the species, that is taken as 100%).

Table S2. Identification of selected features by MS/MS fragmentation.

	Precursor Ion				Product Ion			
**Metabolite**	**Registered *m/z*, Da**	**Calculated *m/z*, Da**	**Chemical Formula**	**Ion Type**	**Registered *m/z*, Da**	**Reference *m/z*, Da**	**Chemical Formula**	**Ion Type**
valine	118.0861	118.0862	$C_5H_{11}NO_2$	$[M + H]^+$	102.0571	102.0555*	$C_4H_8NO_2$	$[M]^+$
					101.0607	101.0603*	$C_5H_9O_2$	$[M]^+$
					100.0769	100.0762*	$C_5H_{10}NO$	$[M]^+$
					83.0501	83.0497*	C_5H_7O	$[M]^+$
picolinic acid	124.0392	124.0393	$C_6H_5NO_2$	$[M + H]^+$	106.0293	106.0288*	unknown	unknown
					80.0537	80.0500*	C_5H_6N	$[M]^+$
leucine	132,1024	132.1019	$C_6H_{13}NO_2$	$[M + H]^+$	86.097	86.0969 *	$C_5H_{12}N$	$[M]^+$
glutamate	148.0603	148.0607	$C_5H_9NO_4$	$[M + H]^+$	130.0508	130.056*	$C_5H_7NO_3$	$[M + H]^+$
					102.0556	102.056*	$C_4H_8NO_2$	$[M]^+$
					84.0448	84.045*	C_4H_6NO	$[M]^+$
carnitine	162.1126	162.1125	$C_7H_{15}NO_3$	$[M + H]^+$	103.0402	103.039 *	$C_4H_7O_3$	$[M]^+$
					102.0914	102.0913 *	$C_5H_{12}NO$	$[M]^+$
					85.0312	85.0284 *	$C_4H_4O_2$	$[M + H]^+$
arginine	175.1201	175.1196	$C_6H_{14}N_4O_2$	$[M + H]^+$	158.0943	158.0909*	$C_6H_{12}N_3O_2$	$[M]^+$
					157.1101	157.1089*	$C_6H_{13}N_4O$	$[M]^+$
					130.0985	130.0963*	$C_5H_{13}N_4$	$[M]^+$
					116.0714	116.0707*	$C_5H_{10}NO_2$	$[M]^+$
					114.1032	114.1031*	$C_5H_{12}N_3$	$[M]^+$
					112.0874	112.0875*	$C_5H_{10}N_3$	$[M]^+$
glucose	203.0545	203.0526	$C_6H_{12}O_6$	$[M + Na]^+$	145.0517	145.0501*	$C_6H_9O_4$	$[M]^+$
					85.0289	85.0290*	$C_4H_5O_2$	$[M + H]^+$
tryptophan	205.0969	205.0971	$C_{11}H_{12}N_2O_2$	$[M + H]^+$	188.0722	188.0712*	$C_{11}H_{10}NO_2$	$[M]^+$
					170.0616	170.0606*	$C_{11}H_8NO$	$[M]^+$
					159.0933	159.0922*	$C_{10}H_{11}N_2$	$[M]^+$
					144.0824	144.0813*	$C_{10}H_{10}N$	$[M]^+$
					142.0657	142.0657*	$C_{10}H_8N$	$[M]^+$
					132.082	132.0813*	$C_9H_{10}N$	$[M]^+$
					130.0661	130.0657*	C_9H_8N	$[M]^+$
					118.0657	118.0657*	C_8H_8N	$[M]^+$
trehalose	365.1085	365.1054	$C_{12}H_{22}O_{11}$	$[M + Na]^+$	203.0543	203.0521*	$C_6H_{12}O_6$	$[M + Na]^+$
					185.0397	185.0401*	$C_6H_{10}O_5$	$[M + Na]^+$

Identification was carried out by matching fragmentation spectra (result of MS/MS fragmentation of selected features) to reference fragmentation spectra of metabolites from the public metabolite database (HMDB, METLIN); *m*/z—mass-to-charge ratio. A mass tolerance window—0.005 Da.

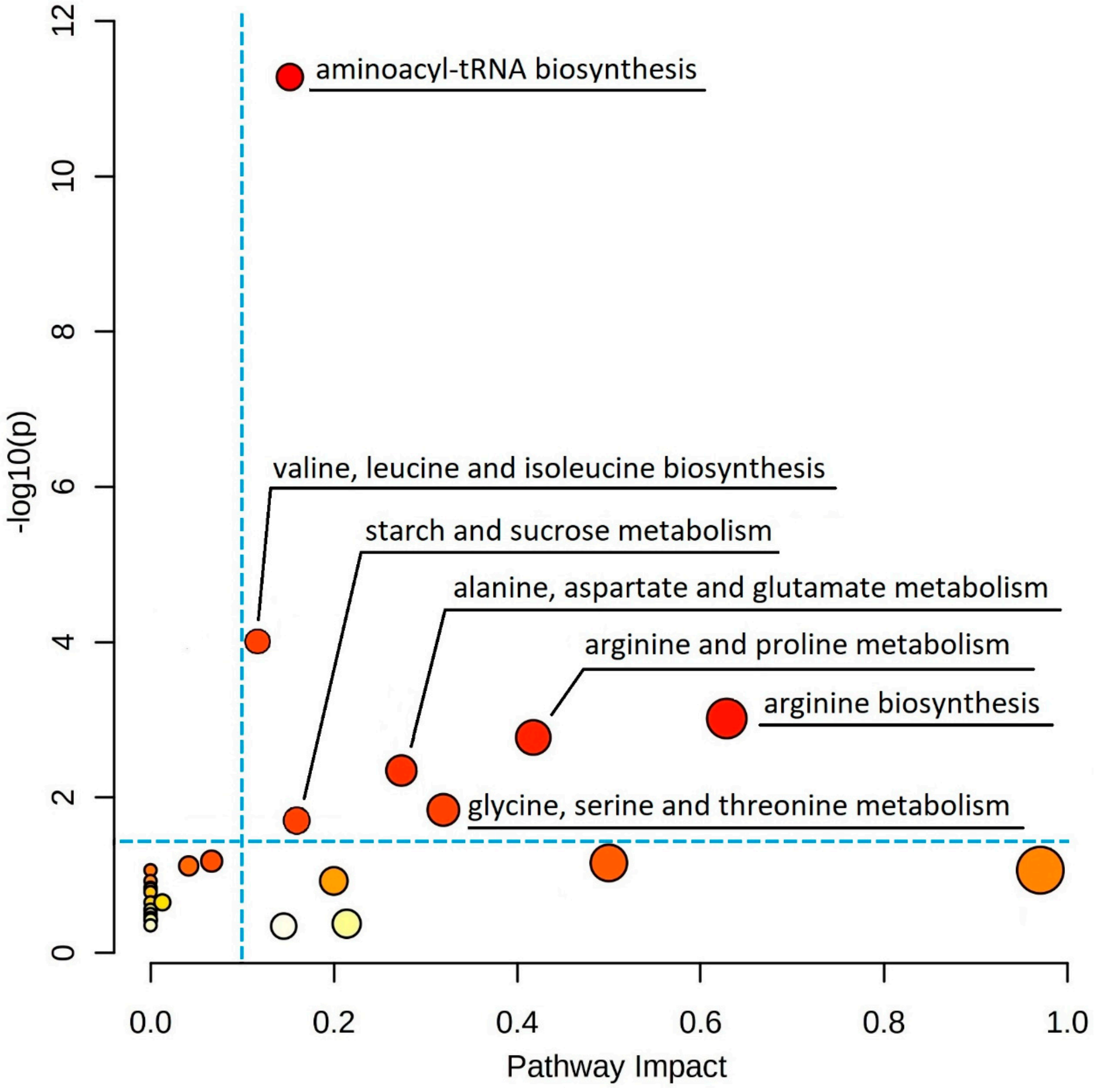

Figure S1. Enriched metabolic pathways detected in the cohort of long-lived fly species.

The y-axis - the negative natural logarithmic value of the original *p*-value. The *p*-value for each metabolic pathway (a group of functional-associated metabolites) computed by pathway enrichment analysis (was used hypergeometric test). The x-axis indicates the pathway impact values (computed by pathway topology analysis) and represents the importance of matched metabolites relative to the specific metabolic pathway. The color of "ball" is associated with negative log(*p*) values; pathway impact values determine its radius. An increase in sizes and darkening of color correlate to the rise of pathway enrichment and pathway impact, respectively (red color- higher significance). The impact values over 0.1 and the *p* values lower than 0.05 (-log(*p*) lower than 1.5) are taken as the thresholds for selecting enriched pathways.

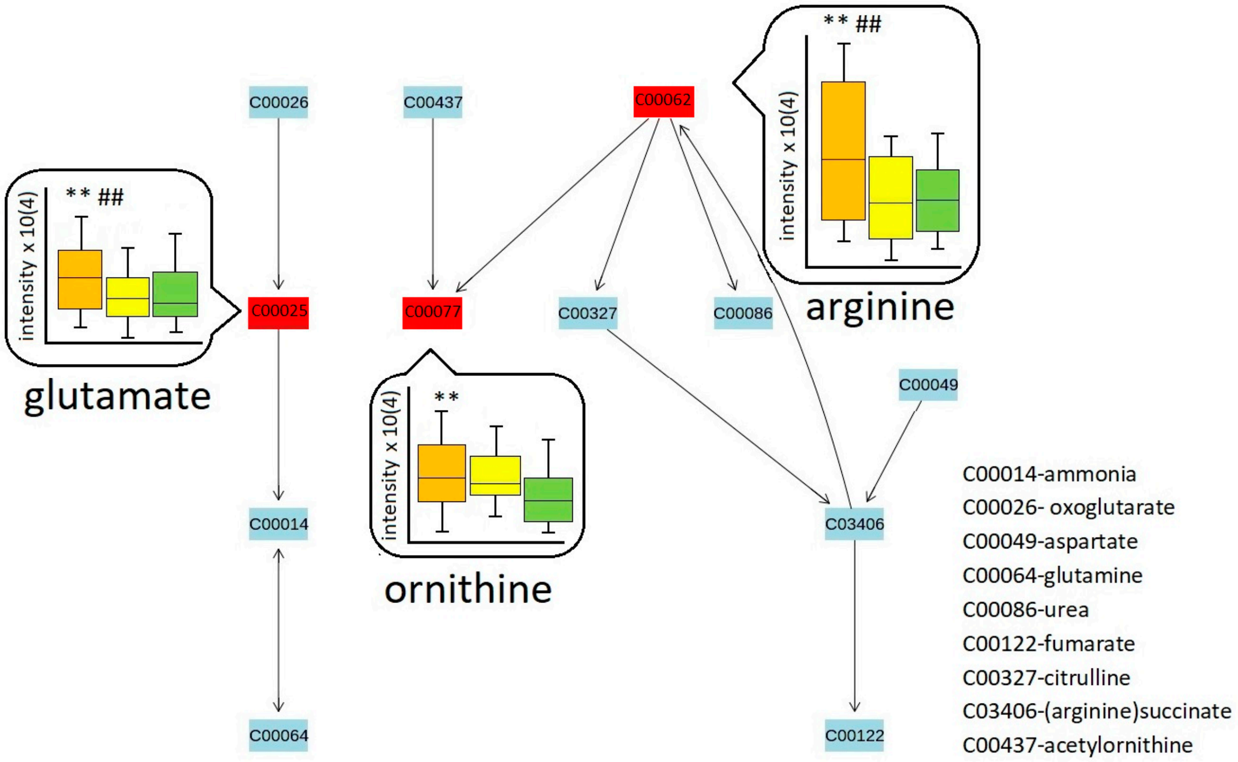

Figure S2. Annotated metabolites on the arginine biosynthesis pathway.

The graphical representation of the arginine biosynthesis pathway is shown. The differentially abundant metabolites are marked by red boxes, other metabolites – by blue boxes. The divergence of the metabolite levels between the cohorts is indicated. The pairwise differences were calculated by the Mann-Whitney *U* test. ** - $p \leq 0.01$ between long-lived and short-lived species; ## - $p \leq 0.01$ between long-lived and medium species. The image was generated using MetaboAnalyst software.

Table S3. Enriched pathways and annotated metabolites.

Pathway name	Annotated metabolites, involved in the pathway	KEGG compound ID
Aminoacyl-tRNA biosynthesis	asparagine	C00152
	arginine	C00062
	glutamate	C00025
	proline	C00148
	tyrosine	C00082
	tryptophan	C00078
	threonine	C00188
	leucine	C00123
	alanine	C00041
	valine	C00183
	serine	C00069
Valine, leucine and isoleucine biosynthesis	leucine	C00123
	valine	C00183
	threonine	C00188
Glycine, serine and threonine metabolism	choline	C00114
	betaine aldehyde	C00576
	threonine	C00188
Arginine biosynthesis	arginine	C00062
	glutamate	C00025
	ornithine	C00077
Arginine and proline metabolism	arginine	C00062
	glutamate	C00025
	proline	C00148
	ornithine	C00077
Starch and sucrose metabolism	glucose	C00031
	trehalose	C01083
Alanine, aspartate and glutamate metabolism	alanine	C00041
	asparagine	C00152
	glutamate	C00025

Table S4. Age-related alterations of putatively annotated metabolites selected by univariate analysis.

№	Metabolite	Ion form	*U* tests		
			**long-lived species "young" vs "mature"**	**medium-lived species "young" vs "mature"**	**short-lived species "young" vs "mature"**
1	alanine	$[M+H]^+$	$6x10^{-3}$	$4x10^{-2}$	$9x10^{-2}$
2	betaine aldehyde	$[M]^+$	$2x10^{-1}$	$4x10^{-1}$	$4x10^{-1}$
3	serine	$[M+H]^+$	$2x10^{-3}$	$2x10^{-1}$	$6x10^{-1}$
4	proline	$[M+H]^+$	$5x10^{-5}$	$4x10^{-3}$	$2x10^{-3}$
5	valine	$[M+H]^+$	$2x10^{-2}$	$2x10^{-4}$	$1x10^{-1}$
6	threonine	$[M+H]^+$	$7x10^{-2}$	$8x10^{-2}$	$2x10^{-1}$
7	picolinic acid	$[M+H]^+$	$2x10^{-1}$	$5x10^{-2}$	$2x10^{-2}$
8	leucine	$[M+H]^+$	$5x10^{-5}$	$9x10^{-3}$	$9x10^{-2}$
9	asparagine	$[M+Na]^+$	$5x10^{-1}$	$9x10^{-1}$	$5x10^{-1}$
10	ornithine	$[M+Na]^+$	$1x10^{-2}$	$5x10^{-2}$	$6x10^{-1}$
11	glutamate	$[M+Na]^+$	$4x10^{-3}$	$3x10^{-2}$	$8x10^{-1}$
12	arginine	$[M+H]^+$	$8x10^{-4}$	$2x10^{-2}$	$3x10^{-2}$
13	tyrosine	$[M+H]^+$	$4x10^{-3}$	$3x10^{-1}$	$6x10^{-1}$
14	tryptophan	$[M+H]^+$	$6x10^{-2}$	$5x10^{-2}$	$7x10^{-1}$
15	choline	$[M]^+$	$5x10^{-2}$	$6x10^{-1}$	$1x10^{-1}$
16	Lyso PE 16:0	$[M+H]^+$	$3x10^{-2}$	$6x10^{-2}$	$5x10^{-1}$
17	Lyso PE 18:1	$[M+H]^+$	$4x10^{-1}$	$1x10^{-1}$	$9x10^{-2}$
18	Lyso PC 16:1	$[M+H]^+$	$3x10^{-1}$	$1x10^{-1}$	$7x10^{-1}$
19	Lyso PC 18:2	$[M+H]^+$	$2x10^{-2}$	$2x10^{-1}$	$4x10^{-1}$
20	Lyso PC 18:1	$[M+H]^+$	$2x10^{-2}$	$8x10^{-1}$	$9x10^{-1}$
21	SM (d33:1)	$[M+H]^+$	$2x10^{-2}$	$5x10^{-1}$	$8x10^{-1}$
22	PC 34:1	$[M+H]^+$	$2x10^{-3}$	$1x10^{-2}$	$5x10^{-1}$
23	PC 36:6	$[M+H]^+$	$3x10^{-2}$	$7x10^{-1}$	$5x10^{-1}$
24	PC 36:2	$[M+H]^+$	$6x10^{-4}$	$7x10^{-1}$	$6x10^{-1}$
25	carnitine	$[M+H]^+$	$9x10^{-4}$	$2x10^{-1}$	$3x10^{-1}$
26	taurine	$[M+Na]^+$	$3x10^{-3}$	$1x10^{-2}$	$3x10^{-2}$
27	glucose	$[M+Na]^+$	$5x10^{-4}$	$2x10^{-3}$	$3x10^{-3}$
28	trehalose	$[M+Na]^+$	$1x10^{-3}$	$1x10^{-3}$	$2x10^{-3}$

In the table are indicated age-related alteration of putatively annotated metabolites selected by univariate analysis. The pairwise differences between two subgroups in each cohort were evaluated by the Mann-Whitney *U* test.

Table S5. Statistical difference of metabolite levels between subgroups of fly cohorts.

№	Metabolite	*H* test	Subgroups of "young" samples *U* tests			*H* test	Subgroups of "mature" samples *U* tests		
			long-lived species vs medium-lived species	long-lived species vs short-lived species	medium-lived species vs short-lived species		long-lived species vs medium-lived species	long-lived species vs short-lived species	medium-lived species vs short-lived species
1	alanine	1×10^{-5}	8×10^{-3}	7×10^{-6}	2×10^{-3}	4×10^{-4}	1×10^{-1}	3×10^{-3}	5×10^{-4}
2	betaine aldehyde	8×10^{-4}	5×10^{-3}	9×10^{-4}	1×10^{-1}	2×10^{-2}	3×10^{-2}	2×10^{-2}	2×10^{-1}
3	serine	5×10^{-6}	3×10^{-2}	3×10^{-6}	2×10^{-4}	1×10^{-2}	9×10^{-1}	6×10^{-3}	8×10^{-3}
4	proline	1×10^{-6}	3×10^{-6}	8×10^{-7}	3×10^{-1}	8×10^{-6}	1×10^{-6}	3×10^{-6}	1×10^{-1}
5	valine	5×10^{-3}	5×10^{-1}	3×10^{-2}	4×10^{-4}	2×10^{-1}	8×10^{-1}	1×10^{-1}	6×10^{-2}
6	threonine	2×10^{-4}	1×10^{-2}	3×10^{-4}	1×10^{-2}	1×10^{-3}	2×10^{-2}	1×10^{-3}	4×10^{-2}
7	picolinic acid	2×10^{-5}	5×10^{-3}	1×10^{-6}	9×10^{-6}	7×10^{-5}	2×10^{-3}	3×10^{-6}	3×10^{-5}
8	leucine	4×10^{-5}	1×10^{-2}	3×10^{-6}	1×10^{-4}	4×10^{-3}	8×10^{-1}	4×10^{-3}	3×10^{-3}
9	asparagine	4×10^{-3}	2×10^{-2}	4×10^{-3}	7×10^{-2}	4×10^{-1}	4×10^{-1}	2×10^{-1}	4×10^{-1}
10	ornithine	9×10^{-5}	5×10^{-1}	5×10^{-5}	7×10^{-5}	7×10^{-2}	9×10^{-1}	2×10^{-2}	9×10^{-2}
11	glutamate	7×10^{-4}	1×10^{-2}	9×10^{-4}	2×10^{-2}	3×10^{-1}	1×10^{-1}	2×10^{-1}	9×10^{-1}
12	arginine	9×10^{-5}	5×10^{-5}	4×10^{-5}	9×10^{-1}	2×10^{-2}	7×10^{-3}	7×10^{-2}	5×10^{-1}
13	tyrosine	2×10^{-5}	7×10^{-3}	2×10^{-6}	4×10^{-1}	1×10^{-2}	6×10^{-2}	2×10^{-3}	7×10^{-1}
14	tryptophan	4×10^{-4}	3×10^{-1}	1×10^{-4}	6×10^{-3}	2×10^{-1}	5×10^{-1}	8×10^{-2}	2×10^{-1}
15	choline	8×10^{-5}	6×10^{-1}	3×10^{-6}	2×10^{-5}	9×10^{-5}	9×10^{-2}	5×10^{-5}	3×10^{-5}
16	Lyso PE 16:0	2×10^{-4}	4×10^{-4}	7×10^{-4}	4×10^{-1}	1×10^{-4}	7×10^{-5}	5×10^{-3}	3×10^{-1}
17	Lyso PE 18:1	2×10^{-4}	1×10^{-2}	8×10^{-4}	5×10^{-5}	3×10^{-4}	2×10^{-2}	7×10^{-5}	2×10^{-3}
18	Lyso PC 16:1	2×10^{-4}	4×10^{-2}	4×10^{-5}	2×10^{-2}	6×10^{-3}	3×10^{-2}	2×10^{-3}	4×10^{-1}
19	Lyso PC 18:2	6×10^{-4}	4×10^{-2}	1×10^{-5}	1×10^{-2}	2×10^{-2}	2×10^{-1}	1×10^{-2}	7×10^{-2}
20	Lyso PC 18:1	1×10^{-6}	1×10^{-6}	1×10^{-7}	7×10^{-4}	1×10^{-4}	2×10^{-3}	6×10^{-5}	2×10^{-2}
21	SM (d33:1)	7×10^{-5}	3×10^{-4}	5×10^{-6}	1×10^{-2}	8×10^{-4}	1×10^{-3}	3×10^{-3}	5×10^{-1}
22	PC 34:1	5×10^{-2}	1×10^{-2}	2×10^{-1}	4×10^{-1}	1×10^{-4}	7×10^{-3}	5×10^{-5}	6×10^{-2}
23	PC 36:6	3×10^{-3}	4×10^{-3}	6×10^{-3}	9×10^{-1}	2×10^{-4}	1×10^{-4}	6×10^{-4}	4×10^{-1}
24	PC 36:2	2×10^{-1}	2×10^{-1}	1×10^{-1}	9×10^{-1}	9×10^{-6}	5×10^{-6}	6×10^{-6}	8×10^{-1}
25	carnitine	6×10^{-4}	6×10^{-4}	2×10^{-5}	1×10^{-3}	3×10^{-5}	1×10^{-1}	7×10^{-6}	8×10^{-4}
26	taurine	4×10^{-4}	6×10^{-2}	1×10^{-4}	5×10^{-3}	2×10^{-2}	8×10^{-1}	9×10^{-3}	2×10^{-2}
27	glucose	9×10^{-5}	8×10^{-5}	1×10^{-4}	1×10^{-3}	2×10^{-5}	6×10^{-3}	3×10^{-5}	2×10^{-4}
28	trehalose	4×10^{-4}	3×10^{-2}	4×10^{-4}	9×10^{-3}	4×10^{-2}	6×10^{-1}	7×10^{-2}	9×10^{-3}

In the table are indicated results of statistical cross-cohort analysis of the formed subgroups (“young” and “mature”). The difference between subgroups of all cohorts (“young” vs “young”, “mature” vs “mature”) was revealed by the Kruskal-Wallis *H* test. The pairwise differences were evaluated by the Mann-Whitney *U* test.

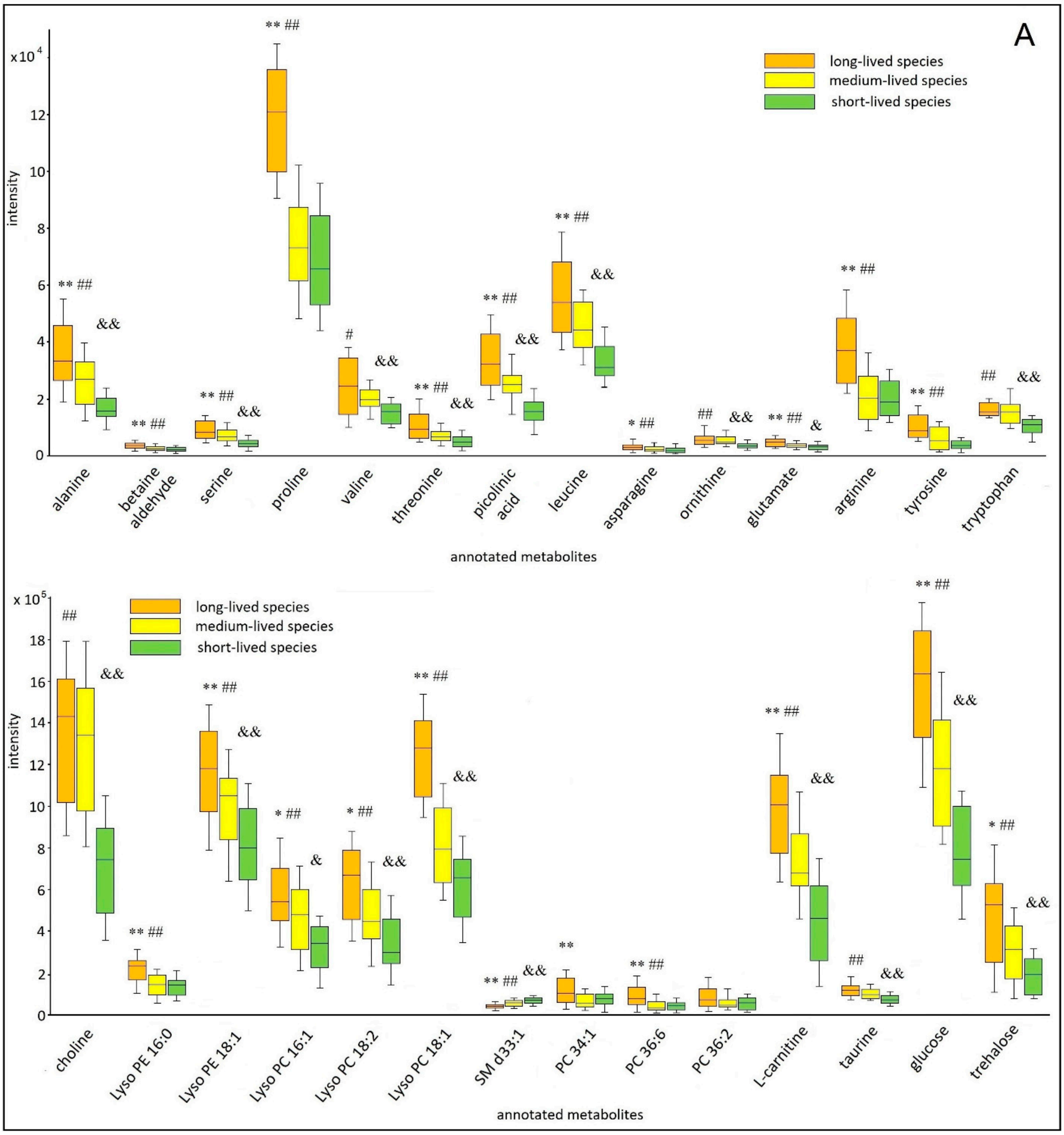
A
long-lived species
medium-lived species
short-lived species
x10^4
intensity
annotated metabolites
alanine
betaine aldehyde
serine
proline
valine
threonine
picolinic acid
leucine
asparagine
ornithine
glutamate
arginine
tyrosine
tryptophan
x 10^5
choline
Lyso PE 16:0
Lyso PE 18:1
Lyso PC 16:1
Lyso PC 18:2
Lyso PC 18:1
SM d33:1
PC 34:1
PC 36:6
PC 36:2
L-carnitine
taurine
glucose
trehalose

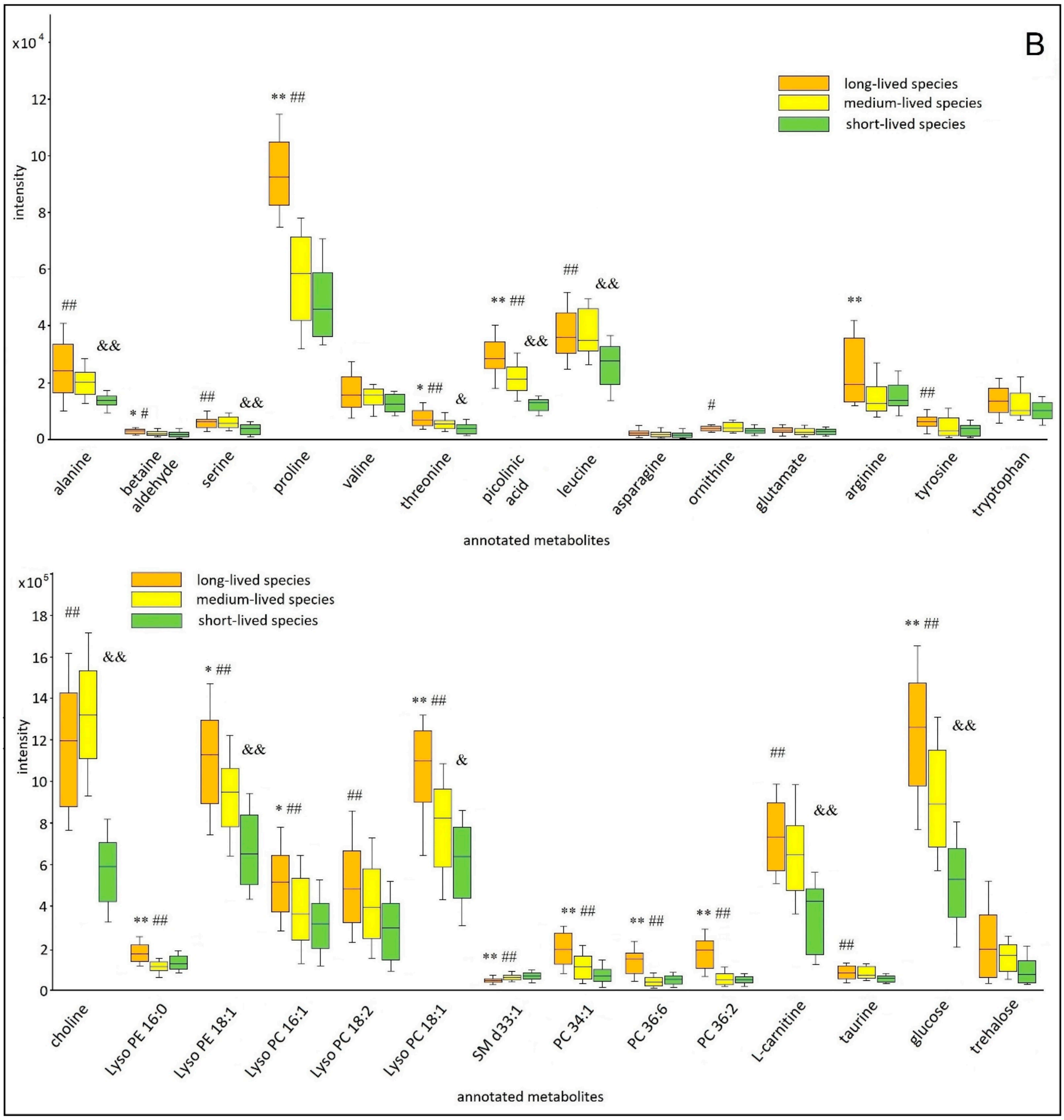

Figure S3. Box and whisker plots of the annotated metabolites of subgroups

The x-axis shows the specific metabolite and the y-axis is the intensity value.The box-whisker plot presents the distribution of normalized intensity values of the annotated metabolites of "young" (A) and "old" (B) subgroups. The top and bottom of the boxes represent the 25% and the 75% percentiles, the 5% and 95% percentiles are indicated as error bars. The median value is indicated by horizontal lines within each box. The outliers were eliminated before the analysis. The results of the pairwise comparisons for the metabolite levels between cohort is displayed. The pairwise differences were calculated by the Mann-Whitney *U* test (* $p \leq 0.05$ (**$p \leq 0.01$) -significant change between long-lived and short-lived species; # $p \leq 0.05$ (## $p \leq 0.01$) - between long-lived and medium species; & $p \leq 0.05$ (&& $p \leq 0.01$)- between medium-lived and short-lived species).
